# Supplementary material for: A host–gut microbial amino acid co-metabolite, p-cresol glucuronide, promotes blood–brain barrier integrity in vivo
Source: Tissue Barriers. 2022 May 20;11(1):2073175. doi: 10.1080/21688370.2022.2073175 (PMC9870004; doi:10.1080/21688370.2022.2073175)
Supplement: Supplemental Material [file KTIB_A_2073175_SM8791.zip › Supplemental Figure Captions.docx]

**Supplemental Figure 1: Treatment with *p*-cresol impairs endothelial barrier integrity *in vitro*. A)** Paracellular permeability of polarised hCMEC/D3 monolayers to a 70 kDa FITC-dextran tracer following 24 h treatment with *p*-cresol (5 µM); data are mean ± s.e.m., n=6. **B)** Trans-endothelial electrical resistance across polarised hCMEC/D3 monolayers following 24 h treatment with *p*-cresol (5 µM); data are mean ± s.e.m., n=6.

**Supplemental Figure 2: Signaling pathway impact analysis (SPIA) for gene expression in mouse brain cells following pCG treatment. A)** SPIA results for all 7702 differentially expressed genes or for the 102 BBB-relevant differentially expressed genes in the CNS of male C57Bl/6 mice 2 h following i.p. injection of 1 mg/kg pCG (n=3 per group). The pathways in red to the right of the thick red line are significant after FWER correction of the global *P* values (pG, obtained by combining the pPERT and pNDE using Fisher's method). The pathways in blue to the right of the thick blue line are significant after FDR correction of the pG values. Numerical labels refer to the KEGG pathway. **B)** Summary of above SPIA results, indicating which KEGG pathways were activated (red) or inhibited (blue).

**Supplemental Figure 3: Expression of TLR4, MD-2 and CD14 by hCMEC/D3 cells.** Typical flow cytometry histogram profiles of hCMEC/D3 cells immunolabelled with **A)** PE-conjugated anti-TLR4, **B)** FITC-conjugated anti-MD-2, or **C)** FITC-conjugated anti-CD14 antibodies.

**Supplemental Figure 4: Exposure of THP-1 cells to LPS dose-dependently increases cell surface CD11b expression. A)** Typical histograms showing a dose-dependent increase in CD11b fluorescence intensity. **B)** Median fluorescence intensities of THP-cell surface CD11b expression in THP-1 cells treated for 24 h with different doses of *Porphyromonas gingivalis* LPS; data are mean ± s.e.m., n=3, **P*<0.05 vs. untreated cells.
