# Supplementary figures and images for: A host–gut microbial amino acid co-metabolite, p-cresol glucuronide, promotes blood–brain barrier integrity in vivo
Source: Tissue Barriers. 2022 May 20;11(1):2073175. doi: 10.1080/21688370.2022.2073175 (PMC9870004; doi:10.1080/21688370.2022.2073175)

Supplemental Figure 1

A

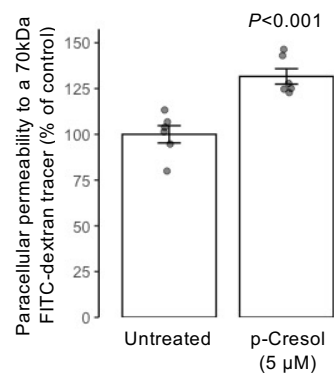

B

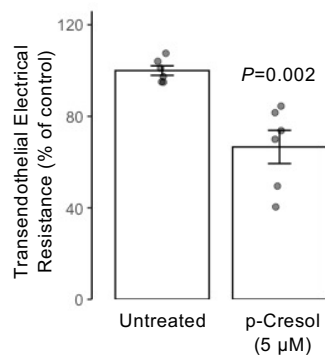

Supplemental Figure 2

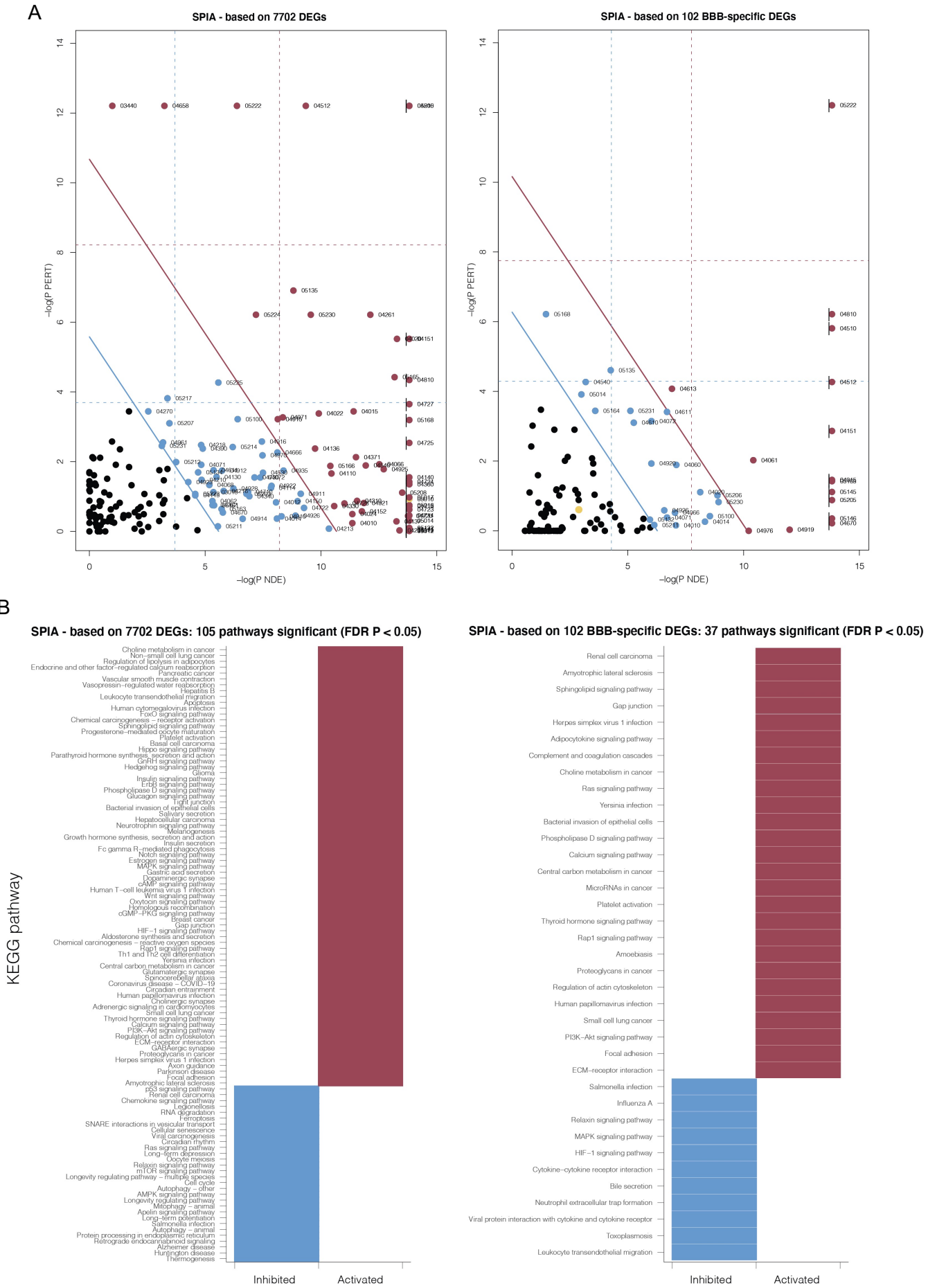

Supplemental Figure 3

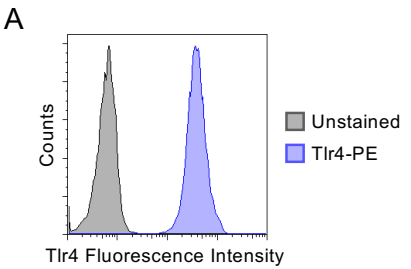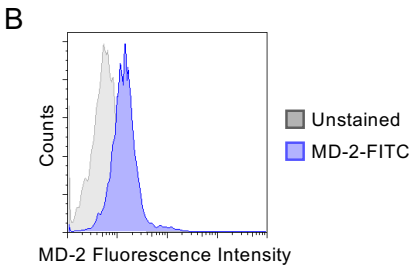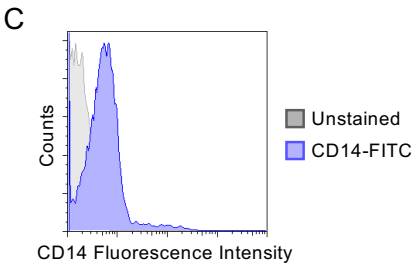

Supplemental Figure 4

A

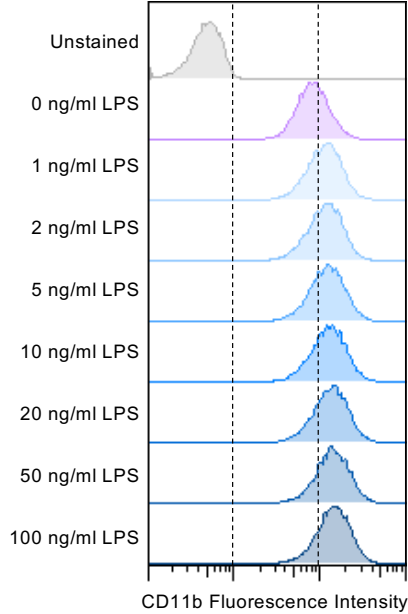

B

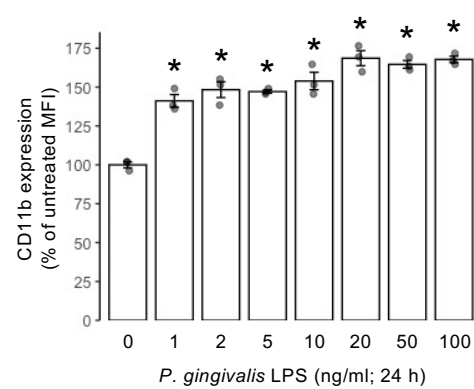

Supplement: Supplemental Material [file KTIB_A_2073175_SM8791.zip › Tissue_barriers_Suppl_Figures.pdf]
